# Low Immune Activation in Early Pregnancy Is Associated With Preterm But Not Small-for-gestational-age Delivery in Women Infected With Human Immunodeficiency Virus Initiating Antiretroviral Therapy in Pregnancy: A Prematurity Immunology in HIV-infected Mothers and their Infants Study (PIMS) Case-control Study in Cape Town, South Africa

**DOI:** 10.1093/cid/ciab151

**Published:** 2021-02-19

**Authors:** Nontlantla Mdletshe, Christina Thobakgale, Thokozile R Malaba, Hlengiwe Madlala, Landon Myer, Daniel M Muema, Polycarp Mogeni, Clive M Gray, Marcus Altfeld, Marie-Louise Newell, Thumbi Ndung’u

**Affiliations:** 1 HIV Pathogenesis Programme, The Doris Duke Medical Research Institute, University of KwaZulu-Natal, Durban, South Africa; 2 School of Pathology, National Institute for Communicable Diseases and the University of the Witwatersrand, Johannesburg, South Africa; 3 Division of Epidemiology and Biostatistics, School of Public Health and Family Medicine, University of Cape Town, Cape Town, South Africa; 4 Africa Health Research Institute, Durban, South Africa; 5 School of Nursing and Public Health, University of KwaZulu-Natal, Durban, South Africa; 6 KwaZulu-Natal Innovation and Sequencing Platform, University of KwaZulu-Natal, Durban, South Africa; 7 Division of Immunology, Institute of Infectious Disease and Molecular Medicine, University of Cape Town, Cape Town, South Africa; 8 Department of Viral Immunology, Heinrich-Pette-Institute, Leibniz Institute for Experimental Virology, Hamburg, Germany; 9 School of Human Development and Health, Faculty of Medicine, University of Southampton, Southampton, United Kingdom; 10 School of Public Health, Faculty of Health Sciences, University of the Witwatersrand, Johannesburg, South Africa; 11 Max Planck Institute for Infection Biology, Berlin, Germany; 12 Ragon Institute of Massachusetts General Hospital, Massachusetts Institute of Technology and Harvard University, Cambridge, Massachusetts, USA; 13 Division of Infection and Immunity, University College London, London, United Kingdom

**Keywords:** HIV, antiretroviral therapy, adverse pregnancy outcomes, monocytes

## Abstract

**Background:**

Mechanisms underlying an association between human immunodeficiency virus (HIV) or antiretroviral therapy (ART) during pregnancy with risk of preterm delivery (PTD) and small-for-gestational-age (SGA) remain unclear. We explored the association between cellular immune activation and PTD or SGA in women with HIV initiating ART during or before pregnancy.

**Methods:**

Women with HIV enrolled at median 15 weeks’ gestation, were analyzed for immune markers, and matched on ART initiation timing (15 women initiated pre- and 15 during pregnancy). There were 30 PTD (delivery <37 weeks), 30 SGA (weight for age ≤10th percentile) cases, and 30 controls (term, weight for gestational age >25th percentile) as outcomes. Lymphocytes, monocytes, and dendritic cell populations and their activation status or functionality were enumerated by flow cytometry.

**Results:**

PTD cases initiating ART in pregnancy showed decreased CD8^+^ T cell, monocyte, and dendritic cell activation; increased classical (CD14^+^CD16^–^) and intermediate (CD14^+^CD16^+^) monocyte frequencies; and decreased inflammatory monocytes (CD14^dim^CD16^+^) compared with SGA cases and term controls (all *P* < .05). Allowing for baseline viral load, the immune markers remained significantly associated with PTD but only in women initiating ART in pregnancy. Lower monocyte activation was predictive of PTD. TLR ligand-induced interferon-α and macrophage inflammatory protein-1β levels in monocytes were significantly lower in PTD women initiating ART in pregnancy.

**Conclusion:**

Low immune activation, skewing toward anti-inflammatory monocytes, and lower monocyte cytokine production in response to TLR ligand stimulation were associated with PTD but not SGA among women initiating ART in, but not before, pregnancy, suggesting immune anergy to microbial stimulation as a possible underlying mechanism for PTD in women initiating ART in pregnancy.

## Teaser

Here, we show that low immune cell activation and lowered cytokine production by monocytes in response to toll-like receptor ligand stimulation in early pregnancy in women initiating antiretroviral therapy in pregnancy is associated with preterm but not small-for-gestational age delivery.

Antiretroviral therapy (ART) improves survival, and prevents mother-to-child human immunodeficiency virus (HIV) transmission [1]. Untreated, advanced HIV disease is associated with adverse birth outcomes [2]; ART in pregnancy has been associated with preterm delivery (PTD), low birth weight, and/or small-for-gestational-age (SGA) infants in some [3–8], but not all [9] studies, possibly driven by specific ART regimen [10, 11]. People with HIV, including pregnant women, are offered ART immediately at HIV diagnosis [12, 13]. Research is needed to inform understanding of potential biological mechanisms underlying any association between HIV or ART and pregnancy outcome [14].

Increased immune activation is required for the maintenance of pregnancy to term, with a physiological shift toward increased peripheral immune cells activation over pregnancy [15–18]. ART reduces systemic immune activation [19–21] that, although demonstrated to be overall clinically beneficial, may also alter immune regulatory pathways linked to immune activation that are essential for normal pregnancy. However, it has also been suggested that excessive systemic immune activation, inflammation at the maternal–fetal interface, and other immune dysfunction may be linked to PTD and other adverse pregnancy outcomes [22–24]. Overall, associations between PTD, SGA, and immunological- and infection-related events are complex, with the exact mechanisms not fully understood [25–28].

We established the Prematurity Immunology in HIV-infected Mothers and their Infants Study (PIMS) in Cape Town, South Africa, to investigate the association between timing of ART initiation (preconception or during pregnancy), immunological parameters, and PTD or SGA [29]. We hypothesized that HIV or ART modulation of immune cell activation status or alteration of immune cells subsets during pregnancy would be associated with PTD or SGA. We focused on T cells, monocytes, and dendritic cells because they play a central immune effector or immunoregulatory role and alterations in their activation status or other perturbations have been reported in HIV infection and pregnancy.

## MATERIALS AND METHODS

PIMS is a prospective cohort study of women with HIV in antenatal care (ANC) at a public sector facility in Cape Town, South Africa [29]. Women with HIV at ≤24 weeks’ gestation, as assessed by ultrasound, were enrolled and followed with 3 study visits for those on ART preconception (stable on ART) at <20 weeks (baseline), 28 and 34 weeks of pregnancy, and an additional study visit 2 weeks after ART initiation for women newly identified as having HIV and initiated on ART at their first ANC visit. At each visit, blood was drawn into sodium heparin tubes (BD Vacutainer, NJ, USA) and peripheral blood mononuclear cells (PBMCs) isolated within 4 hours of blood collection by density gradient centrifugation, counted by the trypan blue method, and stored in liquid nitrogen. For the study presented here, 30 cases of PTD, 30 SGA cases, and 30 appropriate-for-gestational age (AGA)/term controls as outcomes were selected. Controls and cases were matched on timing of ART initiation and analyzed blinded. The median gestational age at enrollment was 15 weeks both for women initiating and stable on ART. PTD was defined as delivery <37 weeks, SGA as weight for gestational age ≤10th percentile, AGA controls were term, with weight for gestational age ≥25th percentile [29]. Baseline information was collected by trained study nurses. CD4 cell counts were closest to the visit on which the sample was taken. Viral RNA was determined for the first 2 study visits.

Ethical clearance was obtained from the Human Research Ethics Committee of the University of Cape Town (reference number 739/2014), the University of Southampton Faculty of Medicine Ethics Committee (reference 12542 PIMS), and the Biomedical Research Ethics Committee of the University of KwaZulu-Natal (reference BE429/15). All participants provided written informed consent.

### Cellular Immunophenotyping and Intracellular Cytokine Staining

Flow cytometry was performed following thawing and counting of PBMCs. A surface stain was performed with antibodies directed against the following antigens: CD3 (Clone: OKT3, BioLegend [BL]), CD4 (Clone: RPA-T4, BL), CD11c (Clone: 3.9, BL), CD123 (Clone: 6H6, BL), CD8 (Clone: RPA-T8 BD Biosciences [BD]), CD56 (Clone: HCD56)/CD19 (Clone: HIB19, BL) (used to exclude natural killer [NK] and B cells, respectively), CD14 (Clone: HCD14-BL), CD16 (Clone: 3G8, BD) (for the identification of monocyte populations depending on the expression of these markers), human leukocyte antigen DR isotype (HLA-DR; Clone: G46-6, BD), CD38 (Clone: HB-7, BL), CD69 (Clone: FN50, BL), and CD86 (Clone: 2331, BL) (markers of activation). Aqua (Life Technologies) viability dye was included for all samples. This was followed by fixation using Perm A (Merck) for 20 minutes in the dark at room temperature.

Cell populations were enumerated, and markers of activation were measured. Samples were acquired on the LSR-II (BD). Cell populations were defined as: CD4^+^ T cells: CD3^+^CD4^+^; activated CD4^+^ T cells: CD3^+^CD4^+^ CD38^+^HLA-DR^+^; CD8^+^ T cells: CD3^+^CD8^+^; activated CD8^+^ T cells: CD3^+^CD8^+^ CD38^+^HLA-DR^+^ (Supplementary Figure 1); monocytes (lineage-HLA-DR^+^CD123^–^CD14^+^) (Supplementary Figure 2), monocytic dendritic cells (mDCs) (lineage-HLA-DR^+^CD11c^+^CD123^–^CD14^–^); and plasmacytoid dendritic cells (pDCs) (lineage-HLA-DR^+^CD11c^–^CD123^+^CD14^–^) (Supplementary Figure 3).

Cytokine production following toll-like receptor (TLR) ligand stimulation was determined by flow cytometry. One and one-half million PBMCs were stimulated with 1 μg/mL lipopolysaccharide (LPS; Merck), 1 μg/mL CL097 (Invivogen), or 500 μM ODN2216 (Invivogen). Unstimulated cells served as negative controls. A total of 5 μg/mL brefeldin A (Sigma) was immediately added to each tube following the addition of TLR ligands to inhibit cellular cytokine release. Intracellular cytokine content of cells was determined after 18 hours of incubation with the respective TLR ligands. All samples were acquired on the LSR II. The percentage of cytokine-producing monocytes, mDCs, and pDCs was determined by FlowJo (Treestar Inc). The gating strategy is shown in Supplementary Figure 4.

### Statistical Analysis

For the analysis of flow cytometry data, FlowJo version 10.5.2 and GraphPad Prism version 5.01 (GraphPad Inc) were used for the graphical representation and nonparametric univariate analyses. Comparisons of paired samples between time points within the same group of individuals were assessed using Wilcoxon matched pairs signed-rank test. Comparisons between different groups of individuals were assessed using Wilcoxon rank-sum test (Mann–Whitney *U* test). To determine the associations of different immune markers and PTD, allowing for baseline viral load, regression models were developed in Stata version 15 (Stata Corp); frequencies of classical and intermediate monocytes were summed because their significant associations with PTD in univariate analyses had similar directionality. To explore the potential use of monocyte activation as a biomarker for PTD, we estimated the sensitivity and specificity of bulk CD14^+^CD86^+^ cells thresholds and used the receiver operating characteristic (ROC) curves to assess its ability to discriminate between PTD and AGA in the logistic regression models. The optimal cutoff for the bulk monocytes expressing CD86 was descriptively determined as the intersection between sensitivity and specificity estimated at various predefined cutoff values.

## RESULTS

Median age was 32 years (interquartile range, 26–36) (Table 1). Of the 90 women, 47 initiated ART prepregnancy (stable on ART) and 43 at first ANC (initiators); most (n = 79, 88%) were on the TDF-3TC-EFV regimen.

**Table 1.** Demographic and Clinical Characteristics of Women Who Initiated ART During or Before Pregnancy

		Initiation Before Pregnancy, N = 47				Initiation During Pregnancy, N = 43			
	Total N = 90	AGA, n = 15	PTD, n = 17	SGA, n = 15	*P* Value	AGA, n = 15	PTD, n = 13	SGA, n = 15	*P* Value
Maternal characteristics									
Age, y: median (IQR)	32 (26–36)	33 (28–35)	36 (32–38)	36 (28–39)	.205	28 (24–34)	26 (25–31)	31 (25–37)	.768
Education, finished high school	32 (35)	5 (33)	5 (29)	5 (33)	.962	7 (47)	6 (46)	4 (27)	.450
Employment status: employed (%)	33 (36)	5 (33)	7 (41)	6 (40)	.889	6 (40)	3 (23)	6 (40)	.565
SES^a^					.741				.860
Lowest	28 (31)	3 (20)	6 (35)	5 (33)		4 (28)	4 (30)	6 (40)	
Medium	30 (33)	6 (40)	5 (30)	5 (33)		5 (35)	5 (38)	4 (26)	
Highest	29 (29)	6 (40)	6 (35)	4 (27)		6 (46)	3 (23)	4 (26)	
Missing	3 (3)	0 (0)	0 (0)	1 (7)		0 (0)	1 (7)	2 (13)	
Obstetric characteristics									
Gravidity, median (IQR)	3 (2–3)	3 (2–4)	3 (2–3.5)	4 (3–4)	.420	2 (2–3)	2 (2–3)	2 (1–2)	.347
Parity, median (IQR)	1(0–2)	1 (1–2)	1 (1–2)	2 (1–2)	.244	1 (0–1)	1 (0–2)	1 (0–2)	.315
Previous preterm^b^: yes	9 (10)	0 (0)	2 (12)	5 (33)	.034	1 (7)	0 (0)	1 (7)	.635
Gestational age at booking/ enrollment, median wk (IQR)	15 (11–18)	13 (9–15)	16 (9–17)	15 (9–18)		14 (12–17)	19 (14–21)	16 (12–18)	
Height, cm: median (IQR)	158 (155.5–162.5)	162 (156–166)	158 (155.5–161)	157 (150.5–163)	.343	160 (156.5–169.5)	158 (155.5–160)	159 (152.5–161.1)	.161
Hemoglobin, g/dL: median (IQR)	11,4 (10–3–12.4)	11 (11.1–12)	11.8 (11–12.7)	11.6 (10.6–12.9)	.283	10.5 (10–12)	11.3 (10.2–11.7)	10.9 (10.1–11.6)	.697
Weight, kg: median (IQR)	70.15 (59.4–83.9)	74.9 (64.30–85.95	76 (67.30–85.45)	61.7 (52.50–74.70)	.126	80,10 (61.70–98.40)	61,35 (58.28–67.48)	64,00 (56.70–92.00)	.413
HIV-associated parameters									
Current ART regimen, self-report					.199				.353
TDF-3TC-EFV	79 (88)	13 (86)	13	13 (86)		14 (93)	11 (84)	15 (100)	
TDF-3TC-NVP	1 (1)	0 (0)	0	0 (0)		1 (7)	0 (0)	0 (0)	
Other NNRTI-based regimen	3 (3)	0 (0)	3	0 (0)		0 (0)	0 (0)	0 (0)	
PI-based regimen	6 (6)	2 (14)	1	2 (14)		0 (0)	1 (8)	0 (0)	
Missing	1 (1)	0 (0)	0 (0)	0 (0)		0 (0)	1 (8)	0 (0)	
CD4 cell count, cells/µL:^c^ median (IQR)	436 (392–573)	416 (353–566)	529 (309–638)	485 (333–584)	.509	368.5 (247–535)	314 (219–457)	493 (283.5–926.5)	.724
Missing	14 (16)	2 (13)	2 (11)	0 (0)		3 (20)	4 (30)	3 (20)	
Viral load, copies/mL, baseline A1, median (IQR)	281 (20–80 000)	20 (20–27)	25 (20–528)	20 (20–65)	.342	21 900 (6120–59 900)	4730 (1300–7950)	2120 (741–18 800)	.681
Viral load, copies/mL, A1.5, median (IQR)	198 (37–431)					431 (275–512)	99 (20–137)	89 (25–307)	.172

Study participants are denoted by number with percentages in parentheses. *P* values denote the comparison across the 3 groups (AGA, SGA, and PTD).

Abbreviations: AGA, appropriate-for-gestational age; ANC, antenatal care; ART, antiretroviral therapy; HIV, human immunodeficiency virus; IQR, interquartile range; NNRTI, non-nucleoside reverse transcriptase inhibitor; SES, socioeconomic status; PI, Protease Inhibitor; PTD, preterm delivery; SGA, small-for-gestational-age.

^a^SES was measured using a composite SES score, based on current employment, housing type, and access to household assets, which was used to categorize women as “high,” “mid,” or “low” SES. A median of these is shown.

^b^All study participants were normotensive. Data were missing for 10 patients initiating therapy during pregnancy and 4 patients who were stable on ART.

^c^CD4 results abstracted from routine records and are the nearest in time to the first ANC visit.

### CD8^+^ But Not CD4^+^ T Cell Activation at Baseline Is Associated With PTD

**Figure 1.**
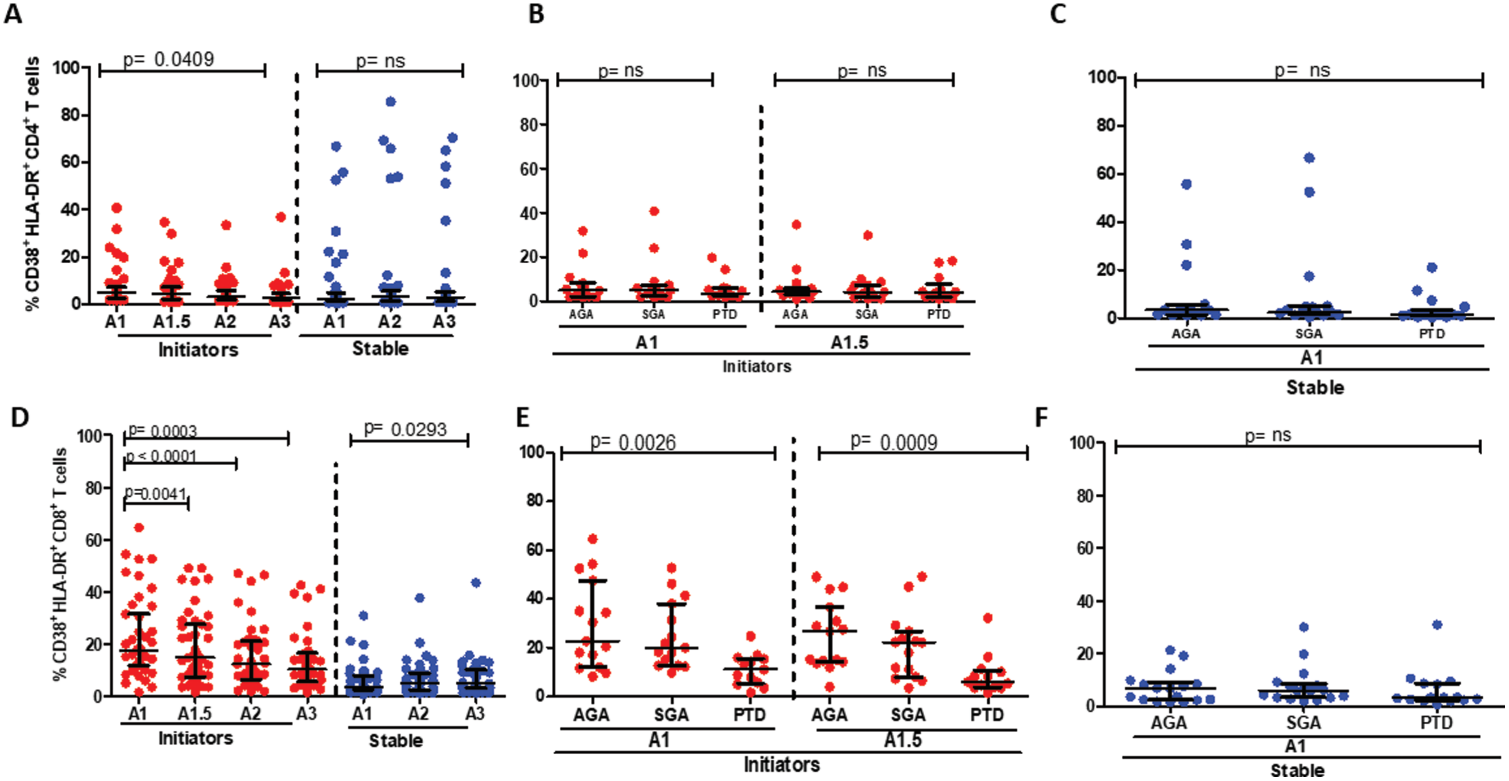
CD4^+^ and CD8^+^ T-cell activation levels in study participants. *A*, CD4^+^ T-cell activation levels for women initiating (red circles) and stable on ART (blue circles) over time and not stratified by birth outcome. *B*, CD4^+^ T-cell activation levels by birth outcomes for women initiating ART (in red) at baseline (A1) and 2 weeks post-ART initiation (A1.5). *C*, CD4^+^ T-cell activation levels by birth outcomes for women stable on ART (blue) at baseline (A1). *D*, CD8^+^ T-cell activation levels for women initiating (red circles) and stable on ART (blue circles) over time. *E*, CD8^+^ T-cell activation levels by birth outcomes for women initiating ART (in red) at baseline (A1) and 2 weeks post-ART initiation (A1.5). *F*, CD8^+^ T-cell activation levels by birth outcomes for women stable on ART (blue) at baseline (A1). Abbreviations: ART, antiretroviral therapy.

The coexpression of CD38 and HLA-DR on CD4^+^ T cells declined significantly between baseline and the last time point for patients initiating ART but not in the ART-stable group (Figure 1A). In initiators at both baseline (ART-naïve) and 2 weeks post-ART initiation, CD4^+^ T-cell activation levels were similar for women with AGA, SGA, or PTD (Figure 1B); likewise, there was no significant difference by pregnancy outcome in women stable on ART (Figure 1C). There was a decrease in CD8^+^ T-cell activation for patients initiating ART, with a lower magnitude but significant decline for participants stable on ART (Figure 1D). Activation levels were lowest for the PTD cases in the initiating group both at baseline (ART-naïve) and 2 weeks thereafter (ART-exposed) compared with the AGA controls and SGA cases (Figure 1E). There was no significant difference in CD8^+^ T-cell activation by outcome for women stable on ART; this was true also for later time points (Figure 1F and data not shown). Interestingly, in the initiators, PTD and SGA cases had significantly lower median viral load compared with controls; lower viral loads were noted for those stable on ART, but there were no significant differences by pregnancy outcome (Supplementary Figure 5). Overall, these data show reduced activation in CD8^+^ but not CD4^+^ T cells for women initiating ART during pregnancy; further, PTD cases who initiate ART in pregnancy have low activation of CD8^+^ T cells; this is partly explained by lower viremia in the PTD group.

### Monocyte Subsets Are Associated With PTD

**Figure 2.**
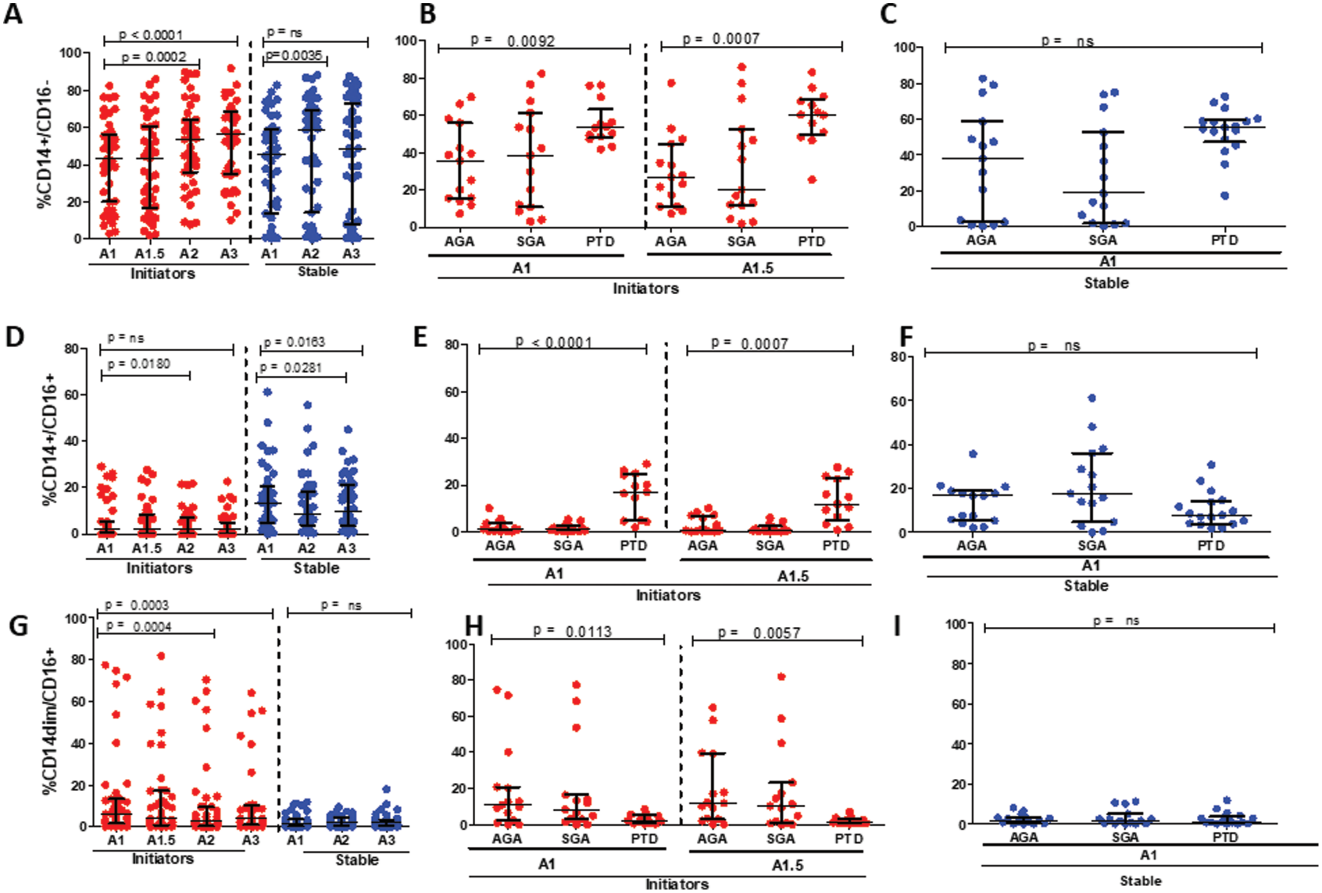
Monocyte frequencies in study participants. *A*, Classical monocyte (CD14^+^CD16^–^) levels for women initiating (red circles) and stable on ART (blue circles) over time and not stratified by birth outcome. *B*, Classical monocyte levels by birth outcome for women initiating ART (in red) at baseline (A1) and 2 weeks post-ART initiation (A1.5). *C*, Classical monocytes levels by birth outcomes for women stable on ART (blue) at baseline (A1). *D*, Intermediate monocyte (CD14^+^CD16^+^) levels for women initiating (red circles) and stable on ART (blue circles) over time and not stratified by birth outcome. *E*, Intermediate monocyte levels by birth outcomes for women initiating ART (in red) baseline (A1) and 2 weeks post-ART initiation (A1.5). *F*, Intermediate monocyte levels by birth outcomes for women stable on ART (blue) at baseline (A1). *G*, Inflammatory monocyte (CD14dimCD16^+^) levels for women initiating (red circles) and stable on ART (blue circles) over time and not stratified by birth outcomes. *H*, Inflammatory monocyte levels by birth outcomes for women initiating ART (in red) baseline (A1) and 2 weeks post-ART initiation (A1.5). *I*, Inflammatory monocyte levels by birth outcomes for women stable on ART (blue) at baseline (A1). Abbreviation: ART, antiretroviral therapy.

Classical monocyte (CD14^+^CD16^–^) frequencies increased significantly over time for initiators, as well as women stable on ART (Figure 2A). Stratified by ART timing and pregnancy outcome, classical monocytes frequencies were consistently higher for PTD cases than SGA cases and AGA controls initiating ART with no significant difference observed for those stable on ART (Figure 2B and 2C). We observed a significant increase of intermediate monocytes frequencies over time in women initiating and stable on ART (Figure 2D). Notably, intermediate monocyte frequencies were higher in PTD than in SGA cases and AGA controls for initiators, both at baseline and 2 weeks post-ART initiation (Figure 2E), but no differences were observed in those stable on ART (Figure 2F). Inflammatory monocyte frequencies decreased significantly over time for the initiators with no change for those stable on ART (Figure 2G). Among initiators, inflammatory monocytes frequencies were lower in PTD than SGA cases and AGA controls (Figure 2H), with no significant differences for those stable on ART (Figure 2I). In summary, classical and intermediate monocyte populations were significantly higher in women initiating ART with subsequent PTD, with reduction in inflammatory monocytes.

### Lower Frequencies of Monocyte, mDC, and pDC Activation (CD86^+^) Are Associated With PTD

**Figure 3.**
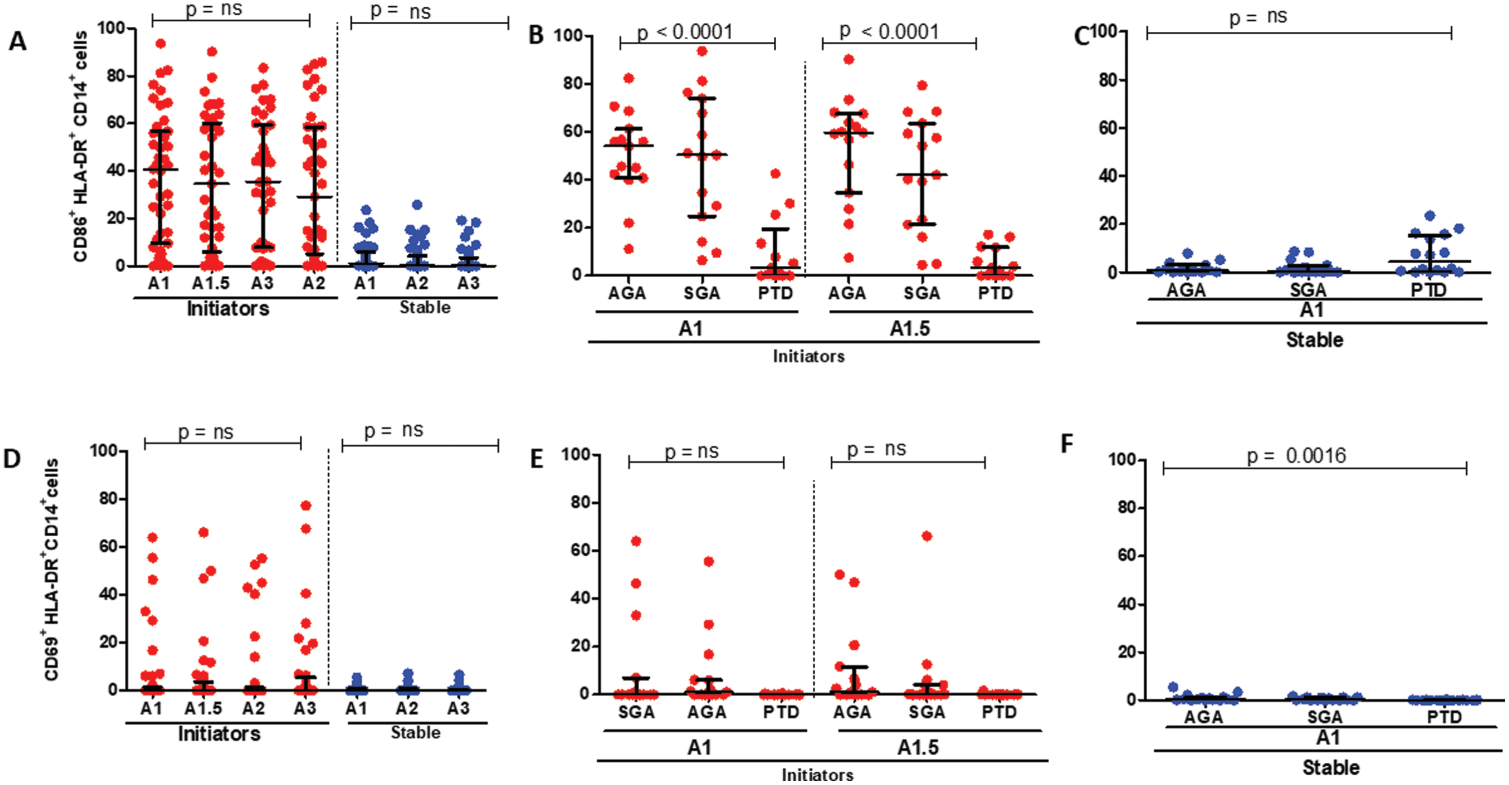
Bulk monocyte activation in study participants. *A*, CD86^+^HLA-DR^+^ expression in bulk monocytes in women initiating (red circles) and stable (blue circles) on ART over time, not stratified by outcome. *B*, CD86^+^HLA-DR^+^ expression outcomes in bulk monocytes for women initiating ART (in red) at baseline (A1) and 2 weeks post-ART initiation (A1.5). *C*, CD86^+^HLA-DR^+^ expression by birth outcomes in bulk monocytes for women stable on ART (blue) at baseline (A1). *D*, CD69^+^HLA-DR^+^ expression in bulk monocytes in women initiating (red circles) and stable (blue circles) on ART over time. *E*, CD69^+^HLA-DR^+^ expression outcomes in bulk monocytes for women initiating ART (in red) baseline (A1) and 2 weeks post-ART initiation (A1.5). *F*, CD69^+^HLA-DR^+^ expression outcomes in bulk monocytes for women stable on ART (blue) at baseline (A1). Abbreviations: ART, antiretroviral therapy; HLA-DR, human leukocyte antigen DR isotype.

**Figure 4.**
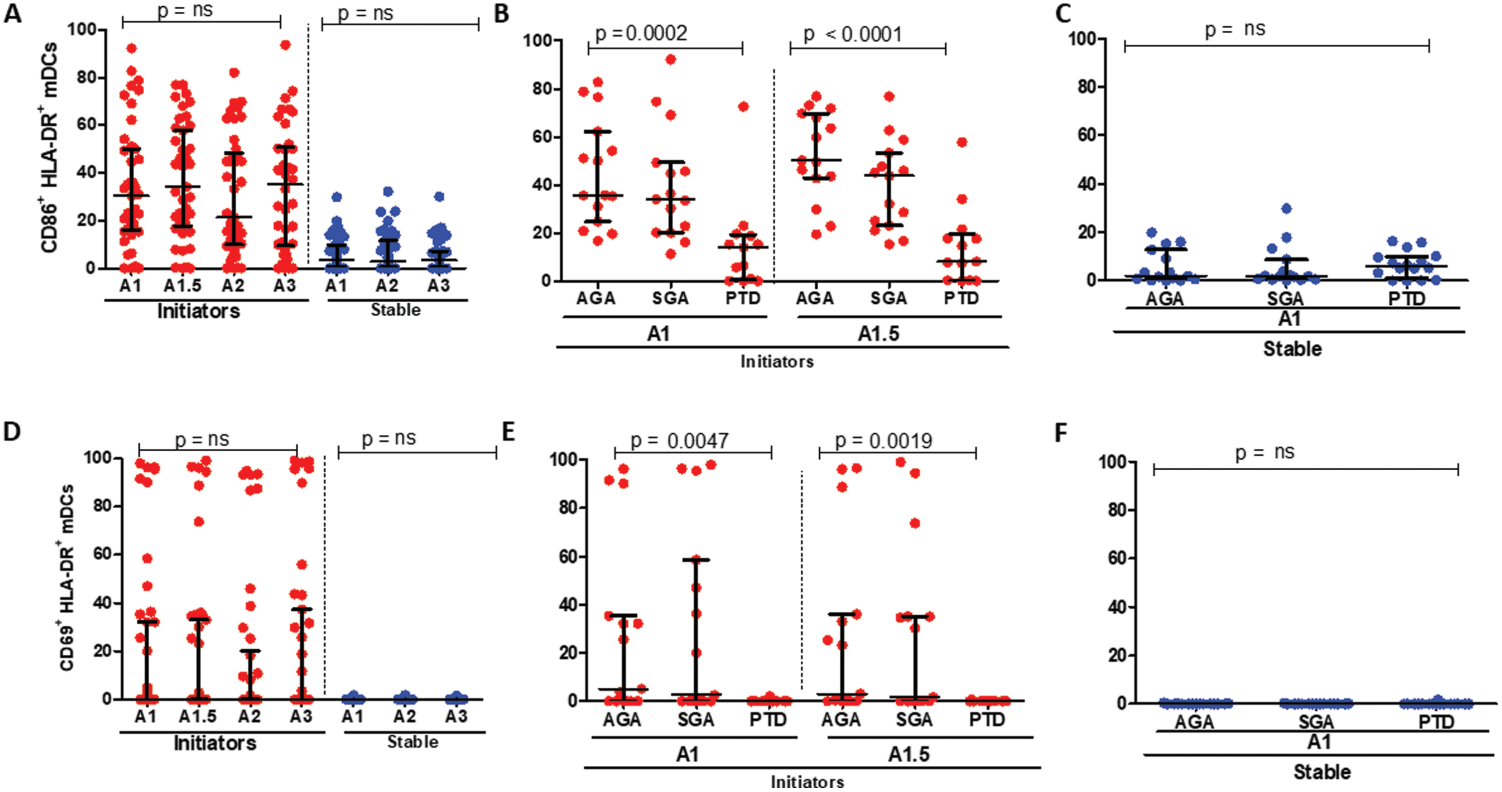
mDC activation in study participants. *A*, CD86^+^HLA-DR^+^ expression in mDCs in women initiating (red circles) and stable (blue circles) on ART over time and not stratified by birth outcome. *B*, CD86^+^HLA-DR^+^ expression by birth outcomes in mDCs for women initiating ART (in red) at baseline (A1) and 2 weeks post-ART initiation (A1.5). *C*, CD86^+^HLA-DR^+^ expression by birth outcomes in mDCs for women stable on ART (blue) at baseline (A1). (D) CD69^+^HLA-DR^+^ expression in mDCs in women initiating (red circles) and stable (blue circles) on ART over time and not stratified by birth outcome. *E*, CD69^+^HLA-DR^+^ expression by birth outcomes in mDCs for women initiating ART (in red) baseline (A1) and 2 weeks post-ART initiation (A1.5). *F*, CD69^+^HLA-DR^+^ expression by birth outcomes in mDCs for women stable on ART (blue) at baseline (A1). Abbreviations: ART, antiretroviral therapy; HLA-DR, human leukocyte antigen DR isotype; mDC, monocytic dendritic cell.

**Figure 5.**
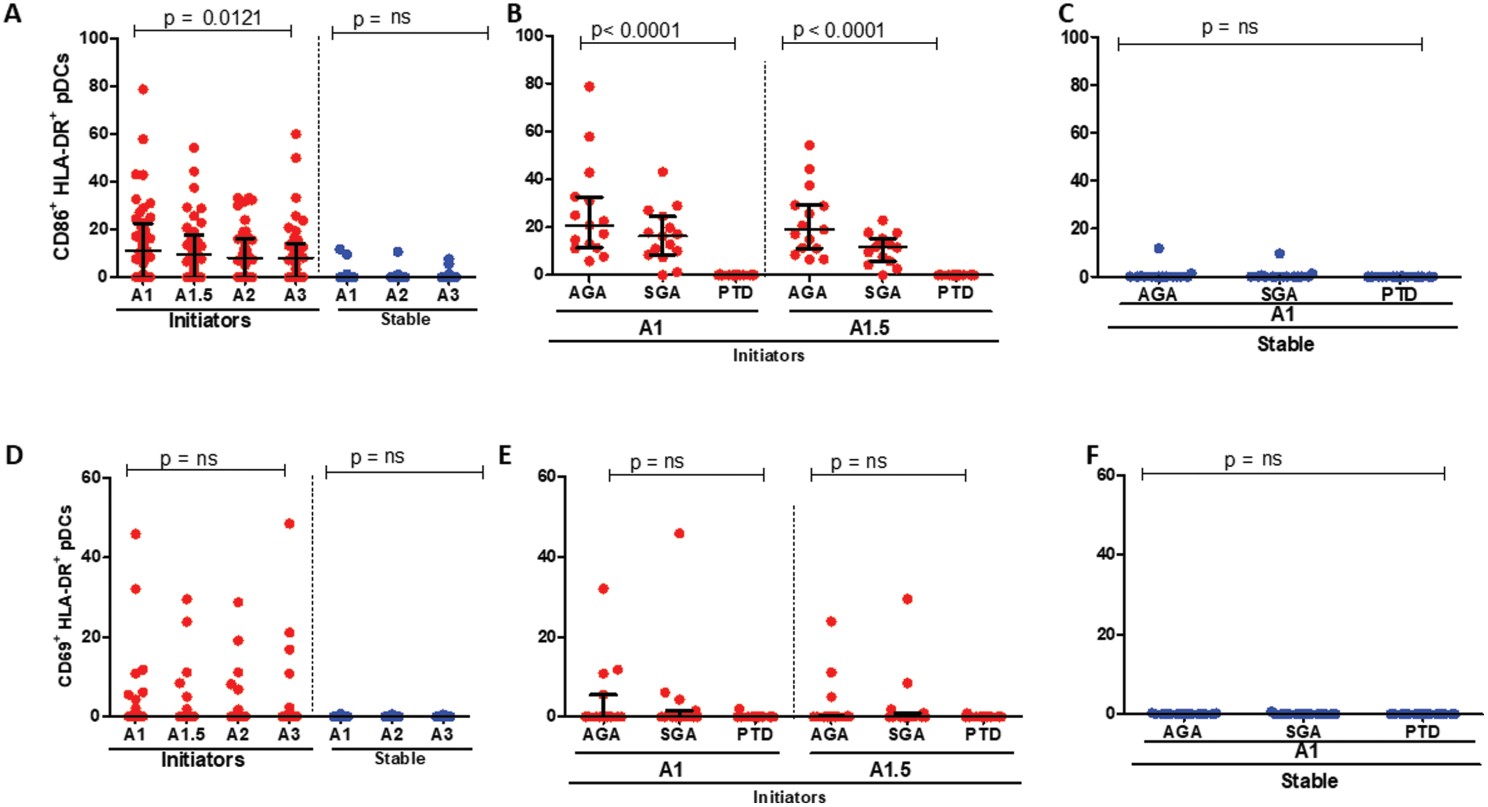
pDC activation in study participants. *A*, CD86^+^HLA-DR^+^ expression in pDCs in women initiating (red circles) and stable (blue circles) on ART over time and not stratified by birth outcome. *B*, CD86^+^HLA-DR^+^ expression by birth outcomes in pDCs for women initiating ART (in red) baseline (A1) and 2 weeks post-ART initiation (A1.5). *C*, CD86^+^HLA-DR^+^ expression by birth outcomes in pDCs for women stable on ART (blue) at baseline (A1). *D*, CD69^+^HLA-DR^+^ expression in pDCs in women initiating (red circles) and stable (blue circles) on ART over time and not stratified by birth outcome. *E*, CD69^+^HLA-DR^+^ expression by birth outcomes in pDCs for women initiating ART (in red) baseline (A1) and 2 weeks post-ART initiation (A1.5). *F*, CD69^+^HLA-DR^+^ expression by birth outcomes in pDCs for women stable on ART (blue) at baseline (A1). Abbreviations: ART, antiretroviral therapy; HLA-DR, human leukocyte antigen DR isotype; pDC, plasmacytoid dendritic cell.

There was no significant change in levels of CD86 and CD69 expression in bulk monocytes over time for both initiators and those stable on ART (Figure 3A and 3D). Stratified by outcome, expression of CD86 on monocytes was significantly lower in PTD cases among initiators (Figure 3B), with no significant differences in those stable on ART (Figure 3C). The expression of CD69 did not differ by birth outcome for patients initiating ART; however, expression was significantly lower in PTD cases for ART-stable participants (Figure 3E and 3F). Levels of CD86 and CD69 expression on mDCs were higher in initiators compared with those stable on ART, with no significant change over time for either parameter (Figure 4A and 4D). CD86 levels were significantly lower in PTD cases than in AGA or SGA for ART initiators with no significant difference in those stable on ART (Figure 4B and 4C); with similar results noted for CD69 expression (Figure 4E and 4F). Expression of CD86 declined significantly over time for patients initiating ART, with no significant decline for the ART-stable participants, whereas the expression of CD69 on pDCs did not differ over time (Figure 5A and 5D). Expression of CD86 was significantly lower for PTD cases in initiators; no differences were noted for CD69 among initiators (Figure 5B and 5E). For the ART-stable group, there were no significant differences in expression of CD86 or CD69 by pregnancy outcome (Figure 5C and 5F). Overall, these data demonstrate low activation in APCs in PTD cases compared with AGA and SGA, especially for women initiating ART in pregnancy at baseline.

We next used logistic regression models to explore whether differences in baseline viral loads confounded the associations between immune activation levels and PTD (Table 2). SGA cases were not included given the small and insignificant univariate differences between AGA controls and SGA cases. Because of multicollinearity between the immune markers, we explored the immune markers separately, with adjustment for viral loads. For women initiating ART in pregnancy, lower activation of bulk monocytes, mDCs, and CD8^+^ T cells, as well as higher levels of classical and intermediate monocytes, remained significantly associated with PTD at baseline even after adjusting for viral loads (Table 2). Thus, we were able to rule out the confounding effects of viral load on immune activation status. Furthermore, we performed a network analysis and confirmed interrelationships between the immune activation markers (CD8^+^ T cell, monocyte, inflammatory monocyte subsets, and mDCs) and their associations with PTD among ART initiators (Supplementary Figure 6). There was no evidence of an association between PTD and fold change in biomarkers following treatment initiation.

**Table 2.** Logistic Regression Analysis Allowing for Baseline HIV RNA: Association Between Immune Activation Markers and PTD in Women Initiating ART at First ANC

		Univariable Analysis		Analysis allowing for HIV RNA	
		OR (95% CI)	*P* Value	OR (95% CI)	*P* Value
1	Bulk CD14^+^ monocyte activation (HLA-DR/CD86)	0.89 (0.83–0.96)	.003	0.89 (0.82–0.97)	.006
	Baseline log HIV RNA			0.29 (0.03–2.9)	.291
2	mDC activation (HLA-DR/CD86)	0.92 (0.87–0.98)	.013	0.93 (0.87–0.99)	.028
	Baseline log HIV RNA			0.67 (0.21–2.14)	.502
3	CD8^+^ T-cell activation (HLA-DR/CD38)	0.86 (0.76–0.98)	.026	0.87 (0.76–1.00)	.045
	Baseline log HIV RNA			0.66 (0.21–2.11)	.486
4	Classical and intermediate monocytes	1.22 (1.03–1.44)	.021	1.20 (1.02–1.42)	.031
	Baseline log HIV RNA			0.62 (0.14–2.74)	.526

The models included PTD cases and AGA controls.

Abbreviations: AGA, appropriate-for-gestational age; ANC, antenatal care; ART, antiretroviral therapy; CI, confidence interval; HIV, human immunodeficiency virus; HLA-DR, human leukocyte antigen DR isotype; mDC, monocytic dendritic cell; OR, odds ratio; PTD, preterm delivery.

**Figure 6.**
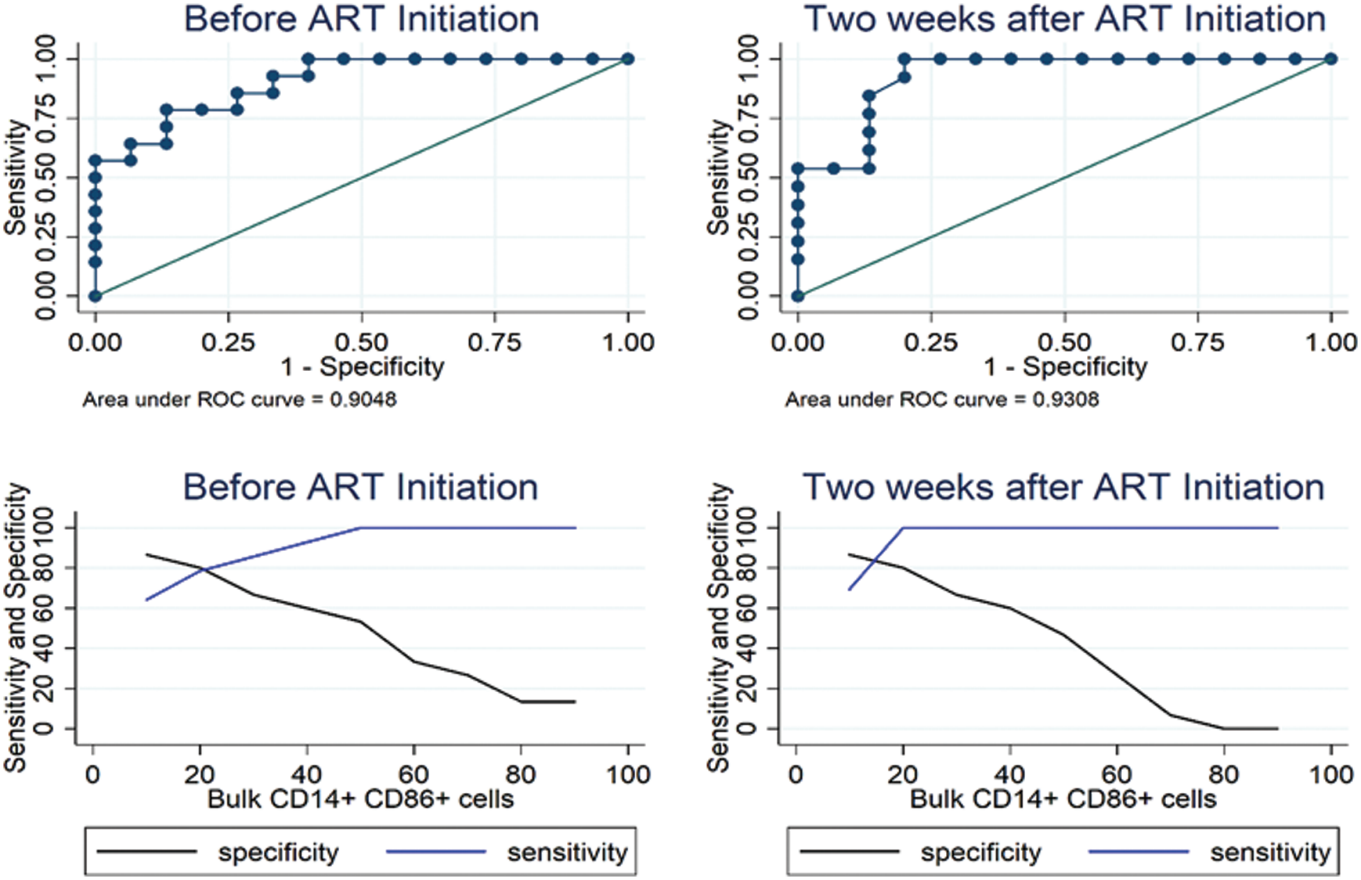
Diagnostic accuracy of bulk monocyte activation for predicting PTD. Diagnostic accuracy of bulk monocyte activation (bulk CD14^+^CD86^+^) for predicting PTD before ART initiation and 2 weeks after ART initiation. Row 1 shows the ROC curves and row 2 shows the sensitivity (blue) and specificity (black) of monocytes at various predefined cutoff points. Abbreviations: ART, antiretroviral therapy; PTD, preterm delivery; ROC, receiver operating characteristic.

Monocyte activation stood out the immunological marker most significantly associated with PTD. We therefore next explored whether monocyte activation was a potential biomarker in early pregnancy to identify women at increased risk of PTD. For women initiating ART in pregnancy, the area under the ROC curve associated with PTD was .905 and .931 at baseline and 2 weeks after ART initiation, respectively. Further, the optimal predictive cutoff for the bulk CD14^+^CD86^+^ cells that optimizes on both sensitivity and specificity was approximately 20% both at ART initiation and 2 weeks later (Figure 6). Overall, these data suggest that monocyte activation is a potential biomarker to identify those at risk of PTD among women with HIV commencing ART in pregnancy.

### Monocyte TLR Ligand-induced Production of Some Cytokines Is Lower in PTD Women Initiating ART in Pregnancy

We hypothesized that lower monocyte activation may reflect senescence or refractoriness to stimulation upon microbial exposure. To address this possibility, we performed intracellular cytokine staining to quantify monocyte production of interferon-α (IFN-α), tumor necrosis factor-α (TNF-α), or macrophage inflammatory protein-1β (MIP-1β) after stimulation with TLR4 (LPS), TLR7/8 (CL097), or TLR9 ligand (ODN2216-CpG) stimulation.

**Figure 7.**
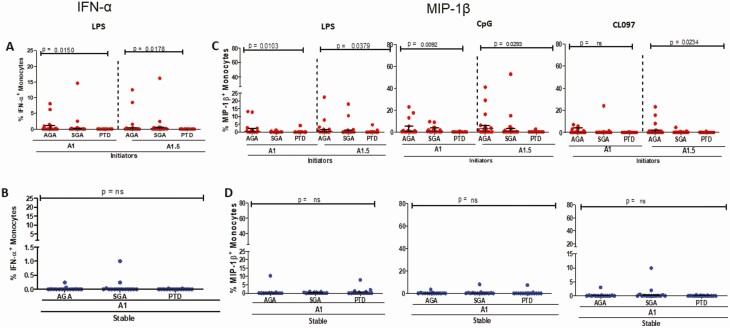
Monocyte IFN-α and MIP-1β expression upon TLR stimulation. *A*, Percent IFN-α expression for each outcome (AGA, SGA, PTD) when stimulated with LPS for patients initiating ART (red circles). *B*, Percent IFN-α expression for each outcome (AGA, SGA, PTD) when stimulated with LPS for patients stable on ART (blue circles). *C*, Percent MIP-1β expression for each outcome (AGA, SGA, PTD) when stimulated with LPS, CpG, and CL097 for patients initiating ART (red circles). *D*, Percent MIP-1β expression for each outcome (AGA, SGA, PTD) when stimulated with LPS, CpG, and CL097 for patients stable on ART (blue circles). Abbreviations: AGA, appropriate-for-gestational age; IFN-α, interferon-α; LPS, lipopolysaccharide; MIP-1, macrophage inflammatory protein-1; PTD, preterm delivery; SGA, small-for-gestational-age; TLR, toll-like receptor.

The percentage of monocytes producing IFN-α in response to all TLR ligands over time did not differ for both those initiating ART and stable on ART (data not shown). When stratified by outcome, patients with the PTD outcome had lower IFN-α expression among ART initiators compared with AGA or SGA (Figure 7A), with no significant difference noted for those stable on ART (Figure 7B). Frequencies of monocytes producing IFN-α in response to TLR7/8 or TLR9 ligands were not different for all outcomes and ART status (Supplementary Figure 7*A* and 7*B*). Monocytes from PTD women produced significantly lower levels of MIP-1β in response to all 3 TLR ligands in women initiating ART, with no such differences noted for women stable on ART (Figure 7C and 7D). Although, as expected, monocytes generally expressed higher levels of TNF-α compared with other cytokines in response to TLR ligand stimulation, there were no significant differences by outcome (Supplementary Figure 8*A* and 8*B*).

## DISCUSSION

We hypothesized that immune activation status, innate immune cell subsets, and their phenotypes or functionality modulated by ART status (initiated pre- or during pregnancy) would be associated with PTD or SGA. In our case-control study, lower CD8^+^ T-cell, monocyte, mDC, and pDC activation (particularly as defined by CD86 but not CD69 expression for the antigen-presenting cells) were all strongly associated with subsequent PTD for women initiating ART in pregnancy but not those who started ART before pregnancy, after allowing for baseline viral load. The association of lower immune activation with PTD in ART initiators was observed before initiation of ART and at 2 weeks post-ART initiation. Further, our findings suggest differences in monocyte subsets by pregnancy outcome with inflammatory monocytes frequencies lower (and vice versa for classical and intermediate monocytes) among ART initiators with subsequent PTD. For most of the immune parameters, women with SGA had similar profiles to control women. ROC curve analysis suggested monocyte activation (CD86 expression) lower than 20% at approximately 15 weeks of gestation as a potential biomarker to identify women at risk for PTD. Interestingly, TLR4-induced monocyte expression of IFN-α and TLR4/TLR-7/8/9–induced expression of MIP-1β was decreased in PTD cases in initiators, suggesting that the reduced immune activation may be indicative of reduced responsiveness to antigen stimulation (immune senescence) as an underlying mechanism. Overall, our findings strongly implicate reduced immune activation as an underlying biomarker for PTD but not SGA.

Notably, lower immune activation was associated with PTD mostly in ART-initiating women and not those stable on ART, suggesting that long-term ART may be leading to correction of the underlying immunological dysfunction. This result further suggests that immune activation is likely only a surrogate for a yet-undetermined immunological dysfunction because women stable on ART with overall reduced immune activation did not have higher incidence of PTD. As expected, initiation of ART rapidly lowered immune activation, with noticeable reduction at 2 weeks after ART initiation but lowered immune activation remained associated with PTD even at that stage, suggesting that the immune defect in ART-naïve individuals associated with PTD is not immediately corrected by ART.

Our study could not definitively identify the immunological dysfunction underlying PTD. However, it has also been suggested that increased inflammation may potentiate adverse pregnancy outcomes, including PTD [30, 31]. Inflammation as an underlying factor for PTD may be indicative of underlying infection as a causative factor for PTD, with the reduced immune activation observed in our study a possible surrogate of reduced potential of immune cells to become activated and respond to infection. Our work highlights the need for additional studies to fully understand the immunological basis for PTD and SGA because many adaptive and innate genes may be involved, both in peripheral and at local reproductive tissue, as shown in previous transcriptomic studies [32]. Our findings of lower immune activation and reduced cytokine production in response to TLR stimulation in pregnant women with subsequent PTD appears consistent with previous reports that PTD infants display a reduced ability to respond to pathogens ex vivo, suggesting a shared immunological phenotype [33, 34]. Overall, our findings suggest that reduced immune activation, which may be linked to reduced immune responsiveness to pathogen insult, could precede PTD and indicate an underlying mechanism, particularly in women initiating ART during pregnancy.

Our study has limitations; first, we could not stratify women by treatment regimen; however, 88% of women were on a TDF-3TC-EFV regimen, the first-line regimen in South Africa. We were also unable to allow for other factors known to be associated with risk of PTD or SGA; however, baseline comparison suggests few differences between the 2 groups, except for age, with women stable on ART being significantly older than those initiating ART. We did not have access to fresh immune cells, but cases and controls were managed to the same protocol.

In conclusion, our study highlights the role of the immune system as one potential mechanistic factor underlying pregnancy outcome. Low immune activation, skewing toward lower levels of inflammatory monocytes, and reduced TLR ligand-induced production of some cytokines by monocytes during pregnancy associated with PTD but not SGA risk in women who initiate ART during pregnancy. Further work is needed to confirm these immunological parameters as potential biomarkers for PTD among women initiating treatment in pregnancy and to explore the exact underlying mechanisms to facilitate better diagnosis and clinical interventions.

## Supplementary Data

Supplementary materials are available at *Clinical Infectious Diseases* online. Consisting of data provided by the authors to benefit the reader, the posted materials are not copyedited and are the sole responsibility of the authors, so questions or comments should be addressed to the corresponding author.

**Figure S1.** Representative gating strategy for the identification of CD4^+^ and CD8^+^ T cell subset activation by flow cytometry. Initial gating was on lymphocytes followed by singlets, exclusion of B cells, NK cells, along with dead cells. Thereafter, CD3^+^ T cells were gated on followed by gating on CD4^+^ and CD8^+^ T cells. The subsequent plots show activation was measured by the expression of HLA-DR and CD38 on CD4^+^ and CD8^+^ T cells, respectively.

**Figure S2.** Identification of blood monocyte subsets by flow cytometry. Gating strategy for identification of monocyte subsets showing firstly gating for single cells and successive exclusion of NK cells and B cells as well as gating on live cells. This was followed by gating for CD3 negative and positive cells. HLA-DR expression was gated on from the CD3 negative cells followed by CD14 vs CD16 to differentiate three (classical, intermediate and inflammatory) monocyte subsets.

**Figure S3.** Representative gating strategy for the identification of bulk CD14, mDC and pDC subset and measurement of activation by flow cytometry. The first dot plot shows forward versus side scatter and all cells were gated on followed by exclusion of NK and B cells, along with dead cells. HLA-DR expression was gated from CD3 negative cells followed by CD14 expression. The subsequent plots were based on the expression of CD86 and CD69 on CD14^+^ cells for monocyte activation and on CD14 negative for CD11c and CD123. Fluorescence minus one (FMO) controls were used to determine the respective gates. Activation in each of the cell populations was based on the expression of CD86 and CD69 markers.

**Figure S4.** Representative gating strategy for the identification of bulk CD14, mDC and pDC subset and measurement of activation by flow cytometry. The first dot plot shows forward versus side scatter and all cells were gated on followed by exclusion of NK and B cells, along with dead cells. HLA-DR expression was gated from CD3 negative cells followed by CD14 expression. The subsequent plots were based on the expression of CD86 and CD69 on CD14^+^ cells for monocyte activation and on CD14 negative for CD11c and CD123. Fluorescence minus one (FMO) controls were used to determine the respective gates. Activation in each of the cell populations was based on the expression of CD86 and CD69 markers.

**Figure S5.** Viral load and CD4 count data for patients initiating and stable on ART. A) Viral loads levels by birth outcomes for women initiating ART (in red) at baseline (A1) and two weeks post ART initiation (A1.5). (B) Viral load levels by birth outcomes for women stable on ART (blue) at baseline (A1). C) CD4 counts by birth outcomes for women initiating ART (in red) at baseline (A1). (F) CD4 counts by birth outcomes for women stable on ART (blue) at baseline (A1). *CD4 count data is missing for 14 patients.

**Figure S6.** Network showing associations between immune parameters and preterm delivery. A) Participants who initiated ART at their first ANC visit. B) Participants who were stable on ART at their first ANC visit. Red lines indicate positive associations. Blue lines indicate negative associations. Associations between immune parameters were assessed by Spearman’s rank order correlation. Associations with premature delivery were assessed by univariate logistic regression.

**Figure S7.** Monocyte IFN-α expression upon TLR stimulation. A). % IFN-α expression for each outcome (AGA, SGA, PTD) when stimulated with CpG and CL097 for patients initiating ART (red circles). B) % IFN-α expression for each outcome (AGA, SGA, PTD) when stimulated with CpG and CL097 for patients stable on ART (blue circles).

**Figure S8.** Monocyte TNF-α expression upon TLR stimulation. A) % TNF-α expression for each outcome (AGA, SGA, PTD) when stimulated with LPS, CpG and CL097 for patients initiating ART (red circles). B) % TNF-α expression for each outcome (AGA, SGA, PTD) when stimulated with LPS, CpG and CL097 for patients stable on ART (blue circles).

ciab151_suppl_Supplementary_Materials

## Notes

***Financial support.*** Research reported in this publication was supported by the Eunice Kennedy Shriver National Institute of Child Health & Human Development of the National Institutes of Health under Award Number R01HD080385. This research was further supported by the President’s Emergency Plan for AIDS Relief (PEPFAR) through the National Institute of Child Health and Human Development (NICHD), grant number 1R01HD074558. Additional funding came from the South African Medical Research Council (Clinician Researcher Development Fund), the National Institutes of Health (NIH) Fogarty International Center Grant #5R25TW009340 and the NIH Office of AIDS Research and the South African Research Chairs Initiative. This study was also supported in part by the South African Department of Science and Technology through the National Research Foundation (South African Research Chairs Initiative), and the Victor Daitz Foundation. Additional funding was provided by the Sub-Saharan African Network for TB/HIV Research Excellence (SANTHE), a Developing Excellence in Leadership, Training and Science in Africa (DELTAS) Africa Initiative (grant # DEL-15-006). The DELTAS Africa Initiative is an independent funding scheme of the African Academy of Sciences (AAS)’s Alliance for Accelerating Excellence in Science in Africa (AESA) and supported by the New Partnership for Africa’s Development Planning and Coordinating Agency (NEPAD Agency) with funding from the Wellcome Trust (grant # 107752/Z/15/Z) and the UK government.

***Potential conflicts of interest.*** None of the authors has conflicts of interest to disclose. All authors: No reported conflicts of interest. All authors have submitted the ICMJE Form for Disclosure of Potential Conflicts of Interest. Conflicts that the editors consider relevant to the content of the manuscript have been disclosed.
