# Supplementary material for: Low Immune Activation in Early Pregnancy Is Associated With Preterm But Not Small-for-gestational-age Delivery in Women Infected With Human Immunodeficiency Virus Initiating Antiretroviral Therapy in Pregnancy: A Prematurity Immunology in HIV-infected Mothers and their Infants Study (PIMS) Case-control Study in Cape Town, South Africa
Source: Clin Infect Dis. 2021 Feb 19;73(12):2205–16. doi: 10.1093/cid/ciab151 (PMC8677566; doi:10.1093/cid/ciab151)
Supplement: ciab151_suppl_Supplementary_Materials [file ciab151_suppl_supplementary_materials.docx]

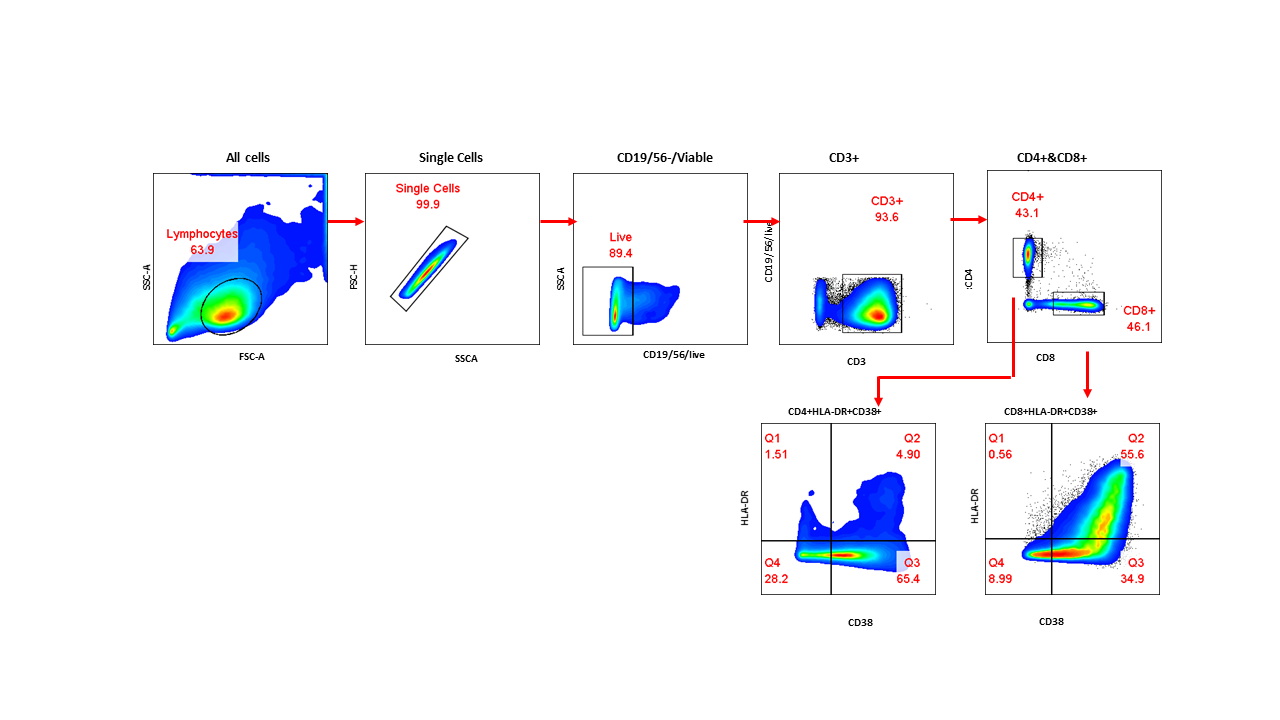


**Figure S1:** Representative gating strategy for the identification of CD4^+^ and CD8^+^ T cell subset activation by flow cytometry.  Initial gating was on lymphocytes followed by singlets, exclusion of B cells, NK cells, along with dead cells. Thereafter, CD3^+^ T cells were gated on followed by gating on CD4^+^ and CD8^+^ T cells. The subsequent plots show activation was measured the by expression of HLA-DR and CD38 on CD4^+^ and CD8^+^ T cells respectively.


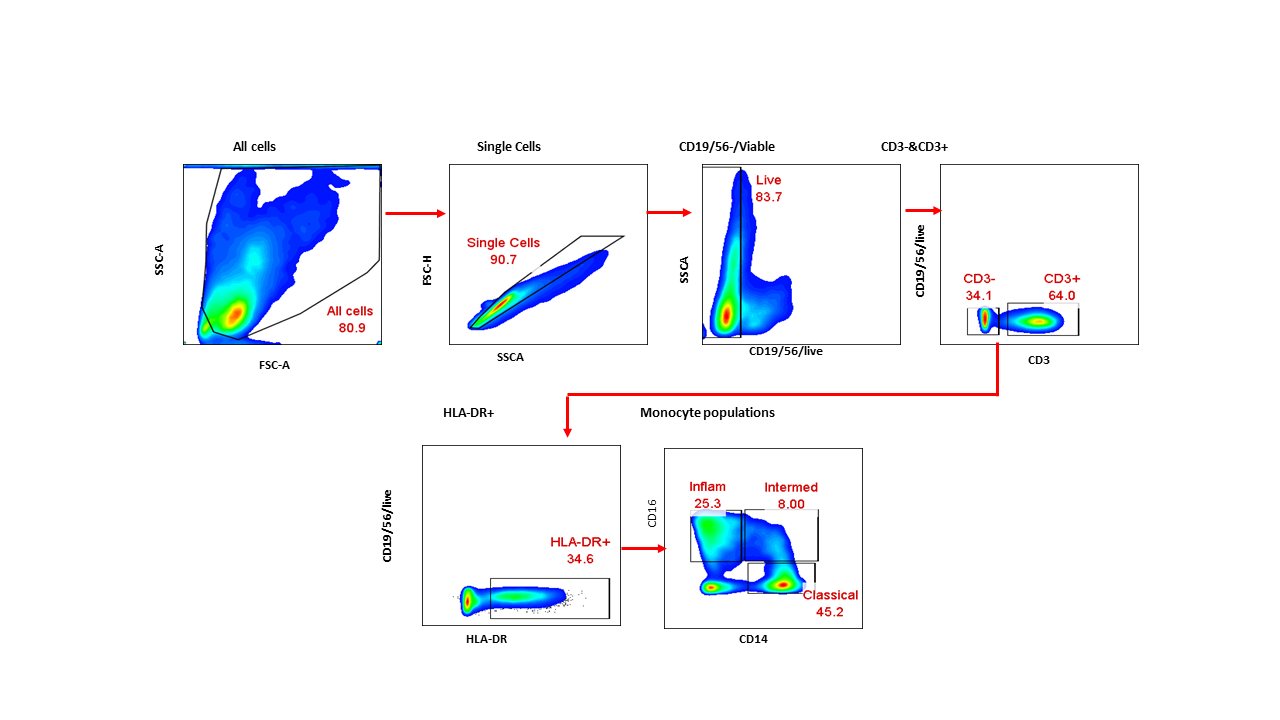


**Figure S2:** Identification of blood monocyte subsets by flow cytometry. Gating strategy for identification of monocyte subsets showing firstly gating for single cells and successive exclusion of NK cells and B cells as well as gating on live cells. This was followed by gating for CD3 negative and positive cells. HLA-DR expression was gated on from the CD3 negative cells followed by CD14 vs CD16 to differentiate three (classical, intermediate and inflammatory) monocyte subsets.


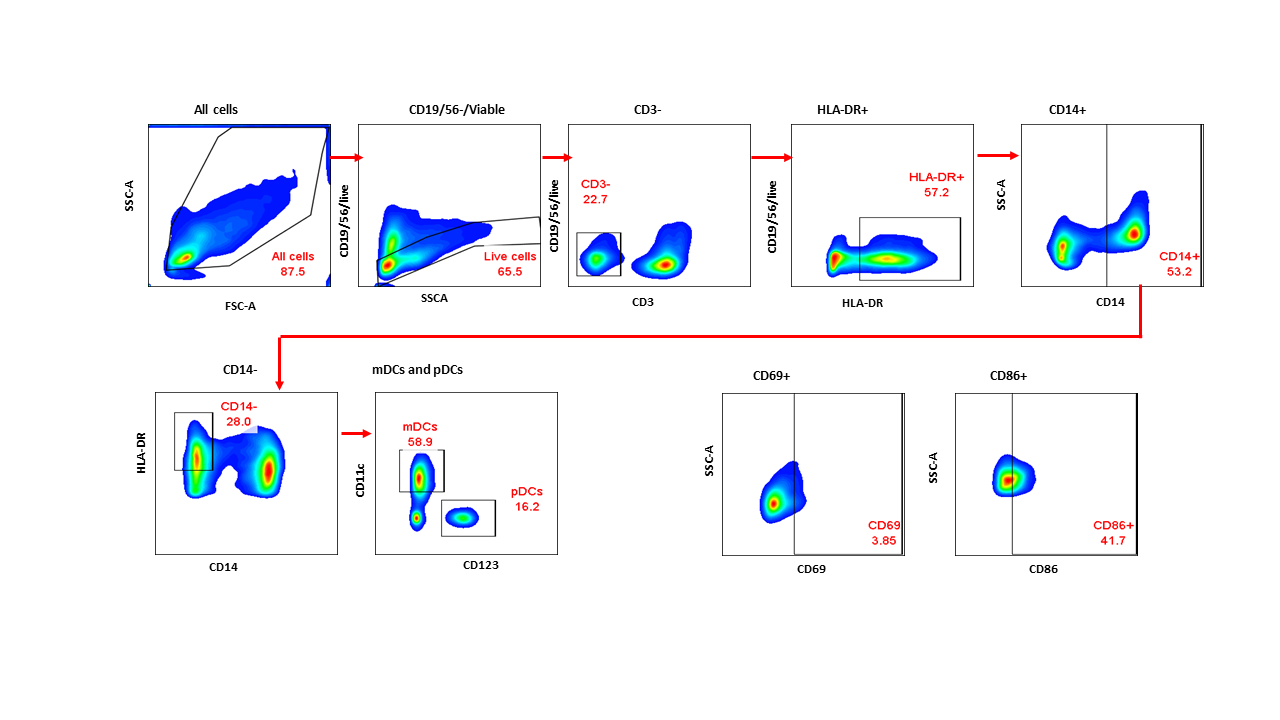


**Figure S3:** Representative gating strategy for the identification of bulk CD14, mDC and pDC subset and measurement of activation by flow cytometry.  The first dot plot shows forward versus side scatter and all cells were gated on followed by exclusion of NK and B cells, along with dead cells. HLA-DR expression was gated from CD3 negative cells followed by CD14 expression. The subsequent plots were based on the expression of CD86 and CD69 on CD14^+^ cells for monocyte activation and on CD14 negative for CD11c and CD123. Fluorescence minus one (FMO) controls were used to determine the respective gates. Activation in each of the cell populations was based on the expression of CD86 and CD69 markers.


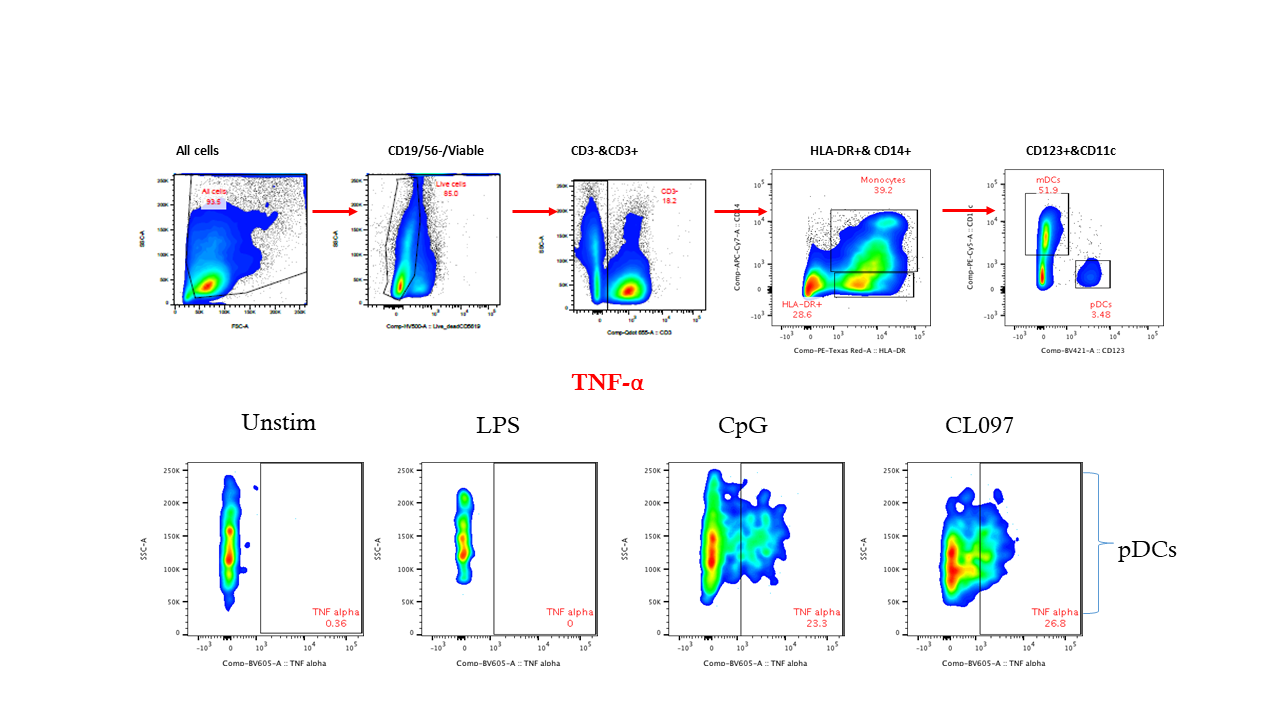


**Figure S4:** Representative gating strategy for the identification of bulk CD14, mDC and pDC subset and measurement of activation by flow cytometry.  The first dot plot shows forward versus side scatter and all cells were gated on followed by exclusion of NK and B cells, along with dead cells. HLA-DR expression was gated from CD3 negative cells followed by CD14 expression. The subsequent plots were based on the expression of CD86 and CD69 on CD14^+^ cells for monocyte activation and on CD14 negative for CD11c and CD123. Fluorescence minus one (FMO) controls were used to determine the respective gates. Activation in each of the cell populations was based on the expression of CD86 and CD69 markers.


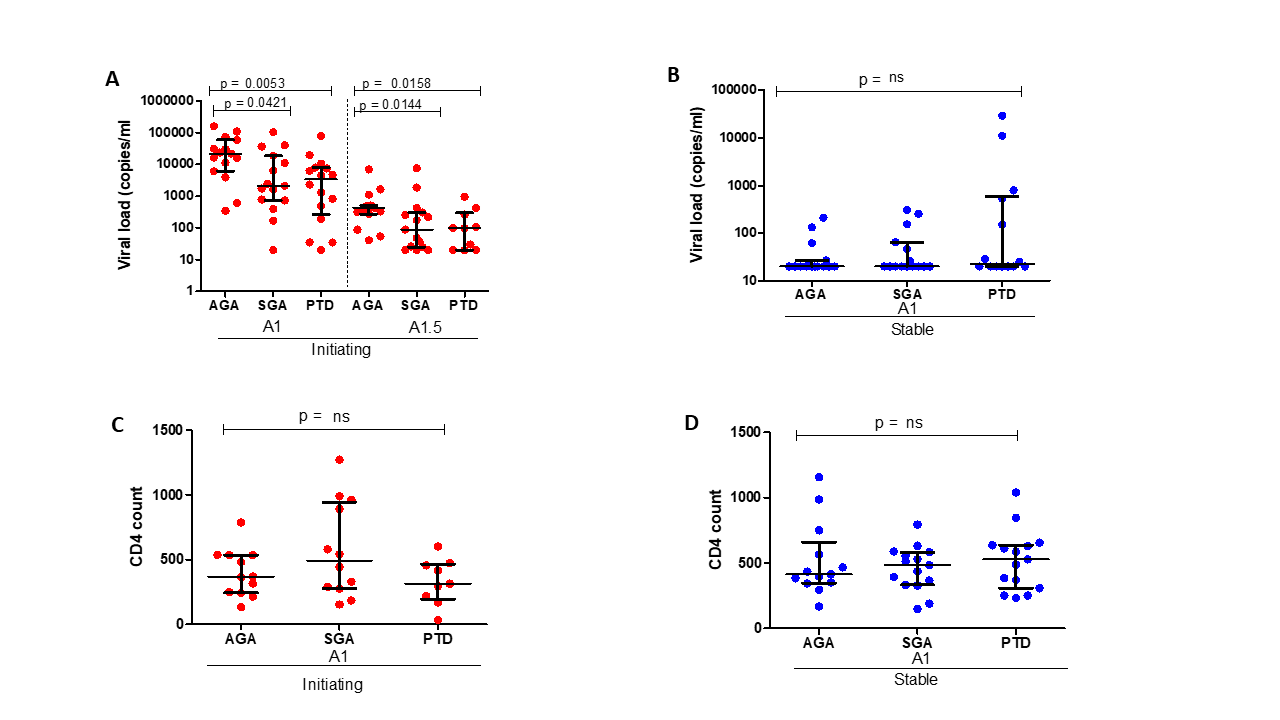


**Figure S5:** Viral load and CD4 count data for patients initiating and stable on ART. A) Viral loads levels by birth outcomes for women initiating ART (in red) at baseline (A1) and two weeks post ART initiation (A1.5). (B) Viral load levels by birth outcomes for women stable on ART (blue) at baseline (A1). C) CD4 counts by birth outcomes for women initiating ART (in red) at baseline (A1). (F) CD4 counts by birth outcomes for women stable on ART (blue) at baseline (A1). *CD4 count data is missing for 14 patients.


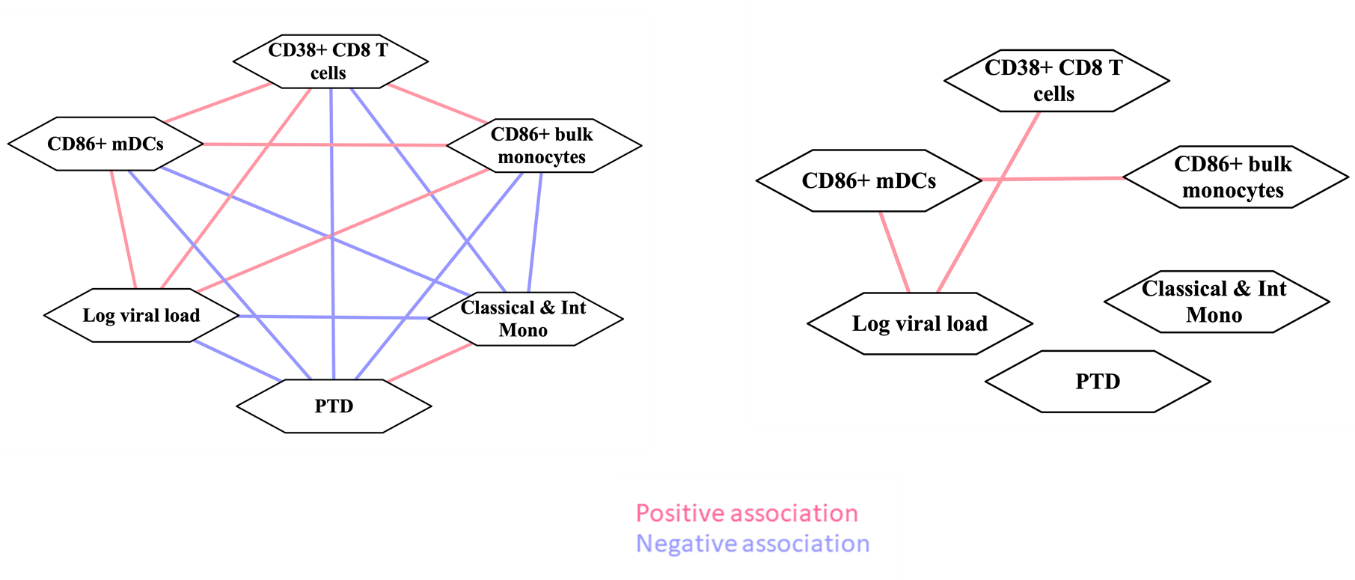


**Figure S6:** Network showing associations between immune parameters and preterm delivery. A) Participants who initiated ART at their first ANC visit. B) Participants who were stable on ART at their first ANC visit. Red lines indicate positive associations. Blue lines indicate negative associations. Associations between immune parameters were assessed by Spearman’s rank order correlation. Associations with premature delivery were assessed by univariate logistic regression.


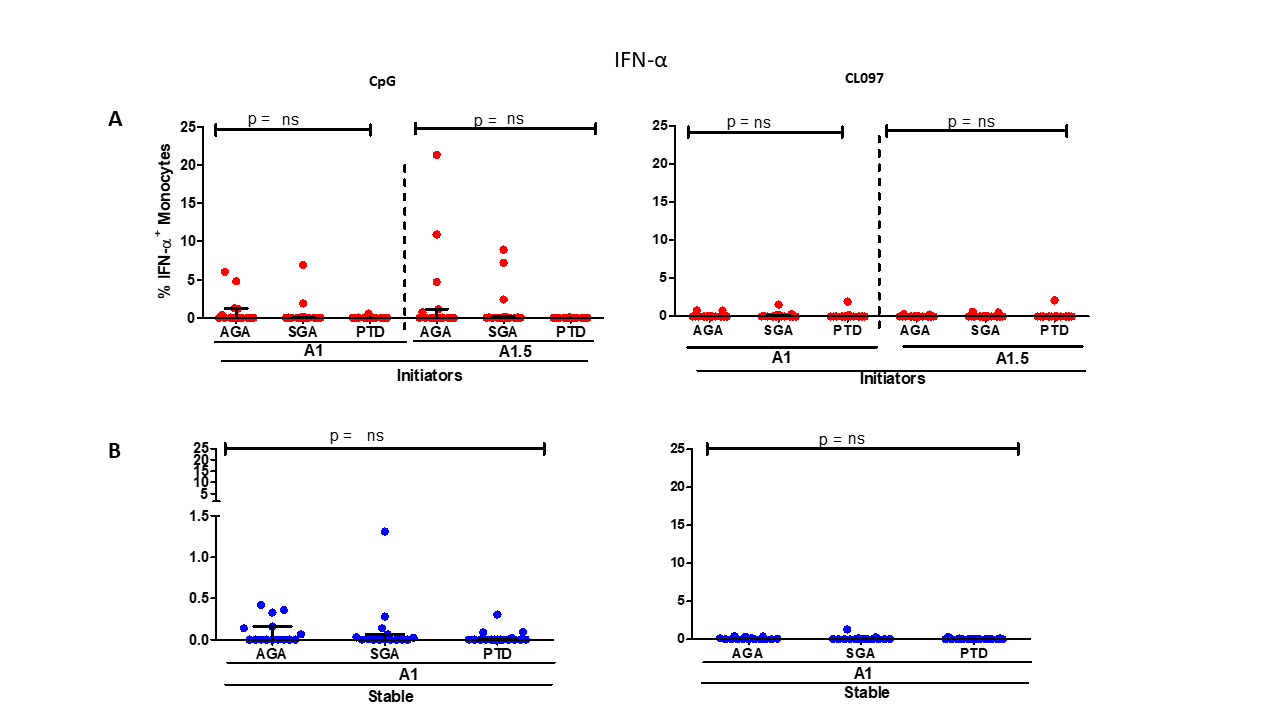


**Figure S7:** Monocyte IFN-α expression upon TLR stimulation. A). % IFN-α expression for each outcome (AGA, SGA, PTD) when stimulated with CpG and CL097 for patients initiating ART (red circles). C) % IFN-α expression for each outcome (AGA, SGA, PTD) when stimulated with CpG and CL097 for patients stable on ART (blue circles).


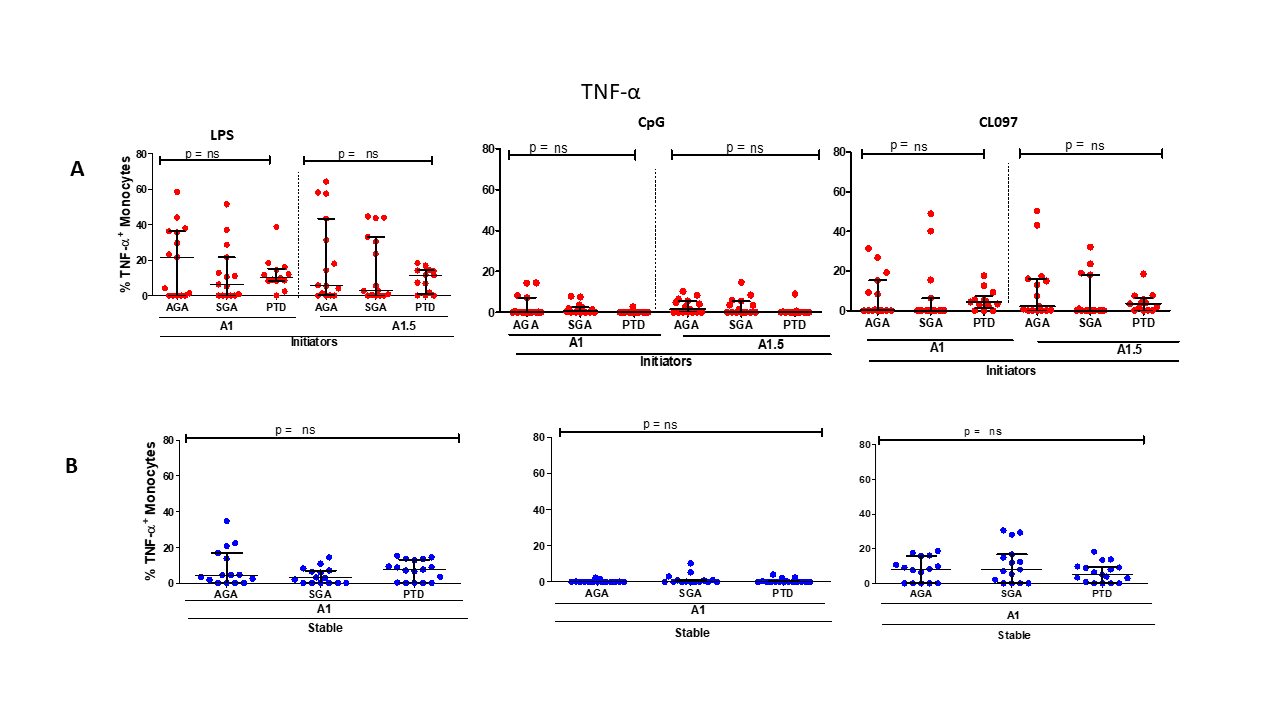


**Figure S8:** Monocyte TNF-α expression upon TLR stimulation. A) % TNF-α expression for each outcome (AGA, SGA, PTD) when stimulated with LPS, CpG and CL097 for patients initiating ART (red circles). B) % TNF-α expression for each outcome (AGA, SGA, PTD) when stimulated with LPS, CpG and CL097 for patients stable on ART (blue circles).
